# Stratifying the Risk of Disease Progression among Surgically Treated Muscle-Invasive Bladder Cancer Eligible for Adjuvant Nivolumab

**DOI:** 10.3390/jcm13185466

**Published:** 2024-09-14

**Authors:** Rocco Simone Flammia, Gabriele Tuderti, Eugenio Bologna, Antonio Minore, Flavia Proietti, Leslie Claire Licari, Riccardo Mastroianni, Alfredo Maria Bove, Umberto Anceschi, Aldo Brassetti, Maria Consiglia Ferriero, Salvatore Guaglianone, Giuseppe Chiacchio, Fabio Calabrò, Costantino Leonardo, Giuseppe Simone

**Affiliations:** 1 Department of Urology, IRCCS “Regina Elena” National Cancer Institute, 00128 Rome, Italy; roccosimone92@gmail.com (R.S.F.); gabriele.tuderti@ifo.it (G.T.); eugenio.bologna@uniroma1.it (E.B.); leslieclaire.licari@uniroma1.it (L.C.L.); riccardo.mastroianni@ifo.it (R.M.); alfredo.bove@ifo.it (A.M.B.); umberto.anceschi@ifo.it (U.A.); aldo.brassetti@ifo.it (A.B.); mariaconsiglia.ferriero@ifo.it (M.C.F.); salvatore.guaglianone@ifo.it (S.G.); gipeppo1@gmail.com (G.C.); puldet@gmail.com (G.S.); 2 Department of Surgery, Sapienza University of Rome, 00161 Rome, Italy; 3 Department of Oncology, IRCCS “Regina Elena” National Cancer Institute, 00128 Rome, Italy; fabio.calabro@ifo.it

**Keywords:** radical cystectomy, adjuvant, chemotherapy, immune check point inhibitors, bladder cancer

## Abstract

**Background:** Check-Mate 274 has demonstrated the disease-free survival (DFS) benefit of adjuvant nivolumab in surgically treated muscle-invasive bladder cancer (MIBC). Since immunotherapy represents an expensive treatment with potential side effects, a better understanding of patient-specific risks of disease progression might be useful for clinicians when weighing the indication for adjuvant nivolumab. **Objective:** To identify the criteria for risk stratification of disease progression among MIBC patients eligible for adjuvant nivolumab. **Materials and methods:** A single-institution, prospectively maintained database was queried to identify patients eligible for adjuvant nivolumab according to Check-Mate 274 criteria. To account for immortal bias, patients who died or were lost to follow-up within 3 months of undergoing a radical cystectomy (RC) were excluded. Kaplan–Meier and Cox regression analyses addressed DFS, defined as the time frame from diagnosis to the first documented recurrence or death from any cause, whichever occurred first. Regression tree analysis was implemented to identify criteria for risk stratification. **Results:** Between 2011 and 2022, 304 patients were identified, with a median follow-up of 50 (IQR 24–72) months. After multivariable adjustment, including NAC as a potential confounder, higher CCI (HR 1.56, 95%CI 1.10–2.21, *p* = 0.013), T stage (HR 2.06, 95%CI 1.01–4.17, *p* = 0.046), N stage (HR 1.73, 95%CI 1.26–2.38, *p* = 0.001) and presence of LVI (HR 1.52, 95%CI 1.07–2.15, *p* = 0.019) increased the risk of disease recurrence or death. Finally, a two-tier classification was developed. Here, five-year DFS rates were 56.1% vs. 18.1 for low vs. high risk (HR: 2.54, 95%CI 1.79–3.62, *p* < 0.001). **Conclusions:** The current risk classification, if externally validated on larger samples, may be useful when weighing the risk and benefit of adjuvant nivolumab treatment and making patients more aware about their disease and about the need for additional treatment after RC.

## 1. Introduction

Muscle-invasive bladder cancer (MIBC) is known to be characterized by one of the highest recurrence rates, even after curative treatment administration [1]; half of the patients suffer from local or distant recurrence within the first 2 years after a radical cystectomy (RC) [2,3,4,5]. Robotic RC is spreading, with promising results in terms of both perioperative morbidity and mortality, even if a survival benefit of this approach has not been proven [6].

Adjuvant chemotherapy plays a significant role in the management of muscle-invasive bladder cancer (MIBC) following radical cystectomy [7]. Its primary objective is to eradicate micrometastatic disease that may not be detectable at the time of surgery but could later manifest as a recurrence. This approach is particularly crucial for patients with high-risk features, such as advanced pathological stage (pT3/pT4) or lymph node involvement (pN+), who are at an elevated risk of recurrence and disease-specific mortality.

Several studies have demonstrated that adjuvant chemotherapy, typically with cisplatin-based regimens, can improve disease-free survival (DFS) and overall survival (OS) in selected MIBC patients [8,9]. The European Association of Urology (EAU) guidelines recommend adjuvant chemotherapy for patients with high-risk pathological features, especially when neoadjuvant chemotherapy was not administered [4]. However, the benefit of adjuvant chemotherapy must be balanced against its potential toxicity, particularly in patients who may have compromised renal function or other comorbidities post-cystectomy.

Recently, Check-Mate 274 demonstrated a disease-free survival benefit for adjuvant nivolumab vs. placebo among high-risk MIBC patients [10]. Specifically, EAU guidelines recommend offering adjuvant nivolumab to high-risk MIBC-affected patients who are not eligible for, or who have declined, adjuvant cisplatin-based chemotherapy or who have already received neoadjuvant cisplatin-based chemotherapy [4]. Since overall survival data are still immature and considering the economic burden deriving from the large-scale use of adjuvant nivolumab, the EMA—in contrast to the FDA—has limited the indication of nivolumab based on PD-L1 expression > 1%. Interestingly, when dissecting data from a pre-planned subgroup analysis, it appears clear that the DFS benefit of nivolumab was more evident among patients with advanced stage at final pathology or those who did not respond to prior neoadjuvant chemotherapy [10]. Analogously, atezolizumab (IMvigor010), despite failing to demonstrate a DFS benefit in the overall cohort, yielded a DFS benefit in patients with circulating tumor cells [11,12], which are known to be an adverse prognostic factor [13].

Taken together, it can be postulated that the efficacy of adjuvant therapies is more pronounced in patients with a greater risk of disease recurrence or death due to the higher likelihood of micrometastasis, which can be effectively managed by systemic therapy. Notably, high-risk MIBC patients represent a heterogenous group [14,15] in terms of DFS trajectories.

Unfortunately, clinical tools to stratify DFS among high-risk MIBC-affected patients are not available. To address this void, we relied on our institutional database; we hypothesized that a refined stratification of DFS among high-risk MIBC patients treated with RC may be accomplished by combining known clinical predictors of recurrence.

## 2. Objective

The primary objective was to identify criteria for risk stratification of disease progression among MIBC patients treated with RC and eligible for adjuvant nivolumab.

## 3. Materials and Methods

### 3.1. Study Design, Setting and Participants

This retrospective cohort study was conducted in accordance with STROBE guidelines (Supplementary File S1). This research study was conducted according to the Declaration of Helsinki’s ethical guidelines. This study was exempt from ethics approval due to its observational nature and due to the retrospective analysis of pseudonymized data.

Within our institution’s prospectively maintained database, we identified BCa patients treated with RC with a high risk of recurrence: specifically, we identified those with pT3, pT4a, or pN+ pathological stage and those who received neoadjuvant cisplatin-based chemotherapy with ypT2 to ypT4a or ypN+ pathological stage.

All patients were treated at the IFO Regina Elena National Cancer Institute, Rome, Italy, from January 2011 to December 2022.

Patients with positive surgical margin, abdominal metastasis diagnosed at the time of surgery, or disease progression/death/censored within 90 days from surgery, as well as those with missing data, were excluded as recommended [16]. The decision to set this timeframe was influenced by the fact that most of our patients underwent the first radiological assessment at 3 months from surgery; thus, patients who died or exhibited recurrence within this time window would be unlikely candidate for adjuvant systemic therapy.

### 3.2. Procedure-Specific Features and Follow-Up

In this study, all patients were initially treated with transurethral resection of the bladder (TURB) confirming the diagnosis of MIBC (cT2 or higher). Following this, a total-body CT scan was implemented for initial staging [17]. Patients were then referred to a dedicated uro-oncologist to be assessed for cisplatin-based NAC eligibility and to be treated accordingly. After that, patients were scheduled for RC, which was performed with open or robot-assisted approaches by two surgeons with strong surgical experience who had previously performed more than 50 operations; the choice of the urinary diversion (ureterocutaneostomy vs. ileal conduit vs. orthotopic neobladder) was considered based on patient age and life expectancy, as well as respecting the patient’s preference following a comprehensive preoperative discussion about the risks and benefits of each option. When pelvic lymph node dissection (PLND) was conducted, an extended template was adopted in all cases [18]. Conversely, in instances of salvage radical cystectomy, which is recognized for providing symptom relief without conferring a survival benefit, PLND was omitted. Patient follow-up was conducted in adherence to the institutional protocols, which were in alignment with the national guidelines prevailing at that time, with the treating physician’s discretion applied as necessary.

### 3.3. Variables

The following baseline characteristics were routinely recorded in our institutional database: age, sex, Charlson comorbidity index (CCI) [19], history of prior intravesical Bacillus of Calmette Guerin (BCG) instillation, and preoperative NAC administration. The pathological characteristics considered were T stage and N stage according to the AJCCth 7th edition [20], number of lymph nodes removed, presence of pure urothelial histology, concomitant CIS, and the presence of necrosis or lymphovascular invasion at final pathology.

### 3.4. Endpoint

The endpoint of interest was disease-free survival, defined as the time between the date of surgery and the date of first recurrence (local recurrence in the urothelial tract, local recurrence outside the urothelial tract, or distant recurrence) or death from any cause, whichever occurred first.

### 3.5. Statistical Analysis

Continuous variables were reported as medians and interquartile ranges (IQRs) and categorical variables as absolute numbers and percentages.

First, univariable Cox regression analysis was conducted to investigate the association between potential predictors, such as CCI, previous BCG, previous NAC, concomitant CIS, pT, pN, presence of LVI or tumor necrosis, and DFS. Subsequently, only statistically significant predictors at the univariable level (*p* < 0.05) were included in the multivariable analysis (MVA), except for NAC. Indeed, NAC was maintained in the MVA to account for potential selection bias and for unexpected prognostic differences between chemotherapy-naïve vs. non-responder patients.

Second, regression tree analysis was implemented to identify criteria for risk stratification in low- vs. high-risk subgroups of disease recurrence or death. A regression tree demonstrates which factors are particularly important in a model or relationship in terms of explanatory power and variance. This process is mathematically identical to certain familiar regression techniques but presents the data in a way that is easily interpreted by those not well versed in statistical analysis. In this way, the regression tree presents a sophisticated snapshot of the relationship between the variables in the data and can be used as a first step in constructing an informative model or a final visualization of important associations. Finally, Kaplan–Meier plots and Cox regression analyses tested the association between this new classification system and DFS.

All tests were two-sided with the level of significance set at *p* < 0.05, and the R software environment for statistical computing and graphics (v.3.4.3) was used for all analyses.

## 4. Results

### 4.1. Baseline Characteristics

Overall, 304 high-risk MIBC-affected patients were identified. The median age was 69 (IQR 62–76) years old; most patients were males (77%) and harbored an age-adjusted CCI > 3 (69.4%). Conversely, only a minority of patients had previously received either BCG instillation (14.1%) or neoadjuvant chemotherapy (25.3%). At final pathology, the majority of patients harbored pure urothelial histology (95.4%), advanced T stage (pT3–4, 90.8%), and LVI (66.1%). Conversely, only 9.2% of patients harbored pT2 stage MIBC at final pathology with concomitant carcinoma in situ (CIS), which was detected in 32.9% of the entire cohort. Furthermore, despite removing a median of 25 (13–34) nodes, pelvic node positivity was detected in a minority of patients (pN1–3 = 32.2%), while the majority of them did not harbor lymph node metastasis or, rarely, had not undergone lymph node dissection. Overall median DFS was 21.0 months (95%CI 15.0 to 35) at three-month landmark analysis. Baseline characteristics are summarized in Table 1.

### 4.2. Cox Regression Model Addressing DFS

At the univariable level, CCI, T stage, N stage, and the presence of LVI all resulted in statistical significance association with disease recurrence or death (all *p* < 0.028, Table 1). Conversely, previous BCG exposure, concomitant CIS, presence of necrosis, and presence of NAC did not show a statistically significant association with disease recurrence or death. After multivariable adjustment, including NAC as a potential confounder, higher CCI (HR 1.56, 95%CI 1.10–2.21, *p* = 0.013), T stage (HR 2.06, 95%CI 1.01–4.17, *p* = 0.046), N stage (HR 1.73, 95%CI 1.26–2.38, *p* = 0.001), and presence of LVI (HR 1.52, 95%CI 1.07–2.15, *p* = 0.019) all increased the risk of disease recurrence and/or death (Table 2).

### 4.3. Regression Tree Analysis

**Figure 1:**
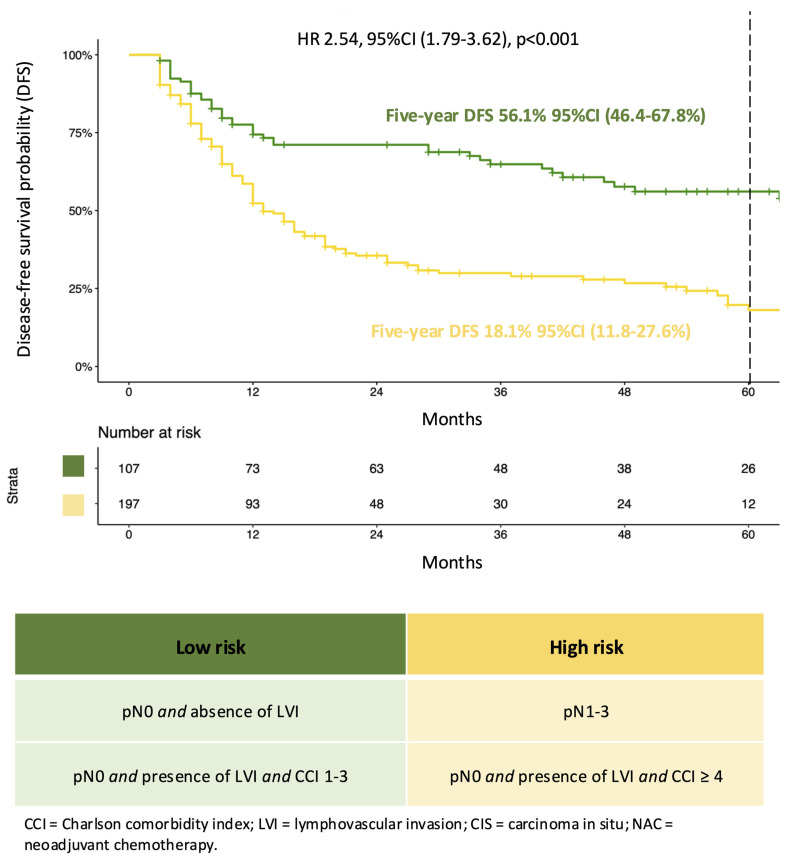
Kaplan–Meier plot and univariable Cox regression analysis addressing disease-free survival according to the new risk classification system applied to RC-treated MIBC patients eligible for adjuvant nivolumab. Additionally, the criteria used to stratify the overall cohort between the two groups are reported.

According to regression tree analysis (Figure 1), four subgroups with progressively worse prognoses were identified: (1) pN0/x, LVI-negative, and any CCI; (2) pN0/x, LVI-positive, and CCI 1–3; (3) pN0/x, LVI-positive, and CCI >3; and (4) pN1–3, any LVI status, and any CCI status. Specifically, Group 2 was not associated with statistically significantly worse prognosis than Group 1 (HR 1.25, 95%CI 0.63–2.48, *p* = 0.5), and similarly, Group 4 did not exhibit a statistically significantly higher risk than Group 3 (HR 1.25, 95%CI 0.88–1.79, 0 = 0.2). In consequence, we decided to combine Groups 1 and 2 and Groups 3 and 4.

### 4.4. New Classification System

A two-tier classification was finally developed based on the low vs. high risk of disease recurrence or death for RC-treated MIBC patients eligible for adjuvant nivolumab (Figure 1). In Kaplan–Meier analysis, five-year DFS rates were 56.1% (46.4–67.8%) vs. 18.1% (95%CI 11.8–27.8%) in low- vs. high-risk patients, respectively. Based on the current classification, high-risk patients exhibit a statistically significant increase in the risk of disease recurrence or death (HR: 2.54, 95%CI 1.79–3.62, *p* < 0.001).

## 5. Discussion

In the current study, we postulated the importance of further stratifying high-risk MIBC patients eligible for adjuvant treatment to guide clinicians in patient management and counseling.

First, we confirmed T stage, N stage, CCI, and LVI as independent predictors of disease recurrence or death. Notably, May et al. externally validated the prognostic role for recurrence of LVI among pT3 BCa patients treated with RC [21] at North American and European institutions (N = 472). More interestingly, Drakaki et al. relied on a contemporary SEER-Medicare cohort including high-risk MIBC (N = 665) and found that AJCC stage IIIB/IVA (HR: 3.2) and CCI > 1 (HR: 1.8) were independent predictors of DFS [22]. However, the former study only included pT3pN0 patients, while the latter lacked LVI data. Conversely, our study not only included the entire spectrum of high-risk MIBC but also collected the most important histological and clinical predictors easily available in clinical practice, thus providing a more comprehensive and detailed analysis.

Second, based on regression tree analysis, we managed to identify two subgroups with statistically different prognoses by pN stage, LVI status, and comorbidity load. Interestingly, patients with either node-positive disease or positive LVI and high comorbidity load exhibited extremely low rates of five-year DFS. Since DFS is a surrogate endpoint for OS [23,24], it can be postulated that an aggressive treatment strategy should be discussed with these patients and started as soon as possible. To date, two potential therapeutic options are available: cis-platinum-based chemotherapy, if not administered in a neoadjuvant setting, and nivolumab in patients who do not respond to cis-platinum-based neoadjuvant chemotherapy or are ineligible for cis-platinum. Additionally, according to a post hoc analysis of the IMvigor trial, atezolizumab may be delivered when a high level of ctDNA is detected [25]. Moreover, urologists should be confident that, in addition to immunotherapy [26], innovative antibody–drug conjugates, such as efortumab–vedotin, are reporting outstanding efficacy in the metastatic setting and thus represent future options in the adjuvant setting [27,28].

Since high-risk patients represent a relevant proportion of MIBC cases treated with RC—and considering the potential biological toxicity and financial burden related to the use of adjuvant systemic therapies such as cis-platinum, immunotherapy, and newer compounds—we firmly believe that a refined stratification of these patients is currently strongly required. Indeed, post hoc analysis of Check-Mate 274 and post hoc analysis of IMVigor clearly demonstrated that adjuvant immunotherapy has greater efficacy when the risk of micrometastasis is higher. Specifically, Galsky et al. showed that including both tumor cell and immune cell PD-L1 expression may better stratify patients who will benefit the most from adjuvant nivolumab administration [29]. Similarly, Powels et al. suggested that ctDNA positivity predicts a benefit with atezolizumab [12].

To the best of our knowledge, although our study relied on well-known prognostic factors, no previous study has combined these variables to obtain a similar result. Unfortunately, due to the retrospective nature of our study, we were not able to provide molecular insights. In this regard, Ben-David et al. found that pre-cystectomy circulating DNA (ctDNA) and detectable ctDNA at the minimal residual disease window (hazard ratio 9.9, 95% CI 2.6–37; *p* < 0.001) were predictive of disease recurrence [30]; thus, they may be targets of clinical interest for adjuvant therapy administration. Interestingly, according to their findings, there is not a clear benefit in terms of recurrence-free survival administering NAC when ctDNA is not preoperatively detected, which paves the way for the definition of new prognostic factors that may be included in daily clinical practice in order to define the best and most patient-tailored approach. Despite the significance of these contributions, these biomarkers, like others, are still under investigation, and time is needed before they will be implemented as cost-effective strategies and before they will be included in the routine evaluation of patients. Meanwhile, our results may help both urologists and patients to gain awareness about disease prognosis, thus improving patient counselling and management. For example, among patients eligible for adjuvant immunotherapies, we may decide to skip adjuvant nivolumab in patients at low risk of disease progression while conducting second-level tests (CTCs, ctDNA+, PD-L1, etc.) in those at high risk, with the ultimate aim of delivering adjuvant therapies to those patients that would most likely benefit from them while avoiding financial burdens and patient toxicity.

Notwithstanding that, our study is not devoid of limitations. First, it represents a retrospective analysis of a single center’s experience, with potential biases in terms of patients’ race or surgeons’ experience with oncological conditions. To minimize these biases, we excluded patients who died or experienced a recurrence within three months of follow-up, which is when the majority of patients received their first postoperative cross-sectional imaging. Thus, this time window represents an optimal “time-zero” which allowed us to exclude the patients that, based on rapidly progressive disease, excessive frailty, or suboptimal/complicated surgery, would not have been eligible for systemic treatment with adjuvant intent. Secondly, we lacked an external validation cohort; thus, future studies should replicate our findings and estimate their predictive accuracy. Third, we did not have access to reliable information on adjuvant chemotherapy status, since our database only specified whether postoperative chemotherapy was delivered, without depicting the clinical scenario of salvage vs. adjuvant chemotherapy.

## 6. Conclusions

According to our findings, risk stratification for disease progression can be made through routinely assessable variables, paving the way for postoperative counseling for patients affected by high-risk MIBC that would be most likely to benefit from adjuvant nivolumab administration.

These results should be confirmed by exploring their weight on the decision-making flowchart when new biomarkers such as ctDNA levels are routinely included.

## Figures and Tables

**Table 1:** Baseline characteristics of the entire cohort of patients eligible for adjuvant nivolumab.

Characteristic	N	Overall, N = 304
**Age** (years)	304	69 (62, 76)
**CCI**	304	4 (3, 6)
**Male sex**	304	234 (77.0%)
**CCI**	304	
1–3		93 (30.6%)
≥4		211 (69.4%)
**Previous BCG**	304	43 (14.1%)
**Previous NAC**	304	77 (25.3%)
**pT stage**	304	
2		28 (9.2%)
3–4		276 (90.8%)
**Node removed**	304	25 (13, 34)
**pN stage**	304	
0/X		206 (67.8%)
1–3		98 (32.2%)
**Histology**	304	
Pure urothelial		290 (95.4%)
**Concomitant CIS**	304	100 (32.9%)
**Presence of necrosis**	304	160 (52.6%)
**Presence of LVI**	304	201 (66.1%)

CCI = Charlson comorbidity index; LVI = lymphovascular invasion; CIS = carcinoma in situ; NAC = neoadjuvant chemotherapy.

**Table 2:** Univariable and multivariable Cox regression analysis addressing disease progression.

	HR	95%CI	*p*-Value	HR	95%CI	*p*-Value
CCI ≥ 4	1.57	1.11–2.21	0.010	1.56	1.10–2.21	0.013
Previous BCG ^‡^	0.80	0.50–1.26	0.333			
Previous NAC ^†^	0.97	0.68–1.38	0.846	1.46	0.98–2.19	0.065
pT 3–4	1.99	1.08–3.67	0.028	2.06	1.01–4.17	0.046
pN 1–3	1.93	1.42–2.63	<0.001	1.73	1.26–2.38	0.001
Presence of LVI	1.86	1.33–2.61	<0.001	1.52	1.07–2.15	0.019
Presence of necrosis ^‡^	1.12	0.83–1.52	0.464			
Concomitant CIS ^‡^	0.79	0.56–1.01	0.162			

CCI = Charlson comorbidity index; LVI = lymphovascular invasion; CIS = carcinoma in situ; NAC = neoadjuvant chemotherapy; HR = hazard ratio; CI = confidence interval. ^†^ NAC was used as a potential cofounder to adjust the multivariable model based on clinical considerations. ^‡^ Previous BCG, presence of necrosis, and concomitant CIS were not considered in the multivariable model based on both a lack of statistical significance and clinical considerations, including lack of evidence for their prognostic role, on top of more relevant risk factors in RC-treated MIBC eligible for adjuvant nivolumab.

## Data Availability

The data are not publicly available due to privacy or ethical restrictions.

## Supplementary Materials

The following supporting information can be downloaded at: https://www.mdpi.com/article/10.3390/jcm13185466/s1.
